# The influence of increased distal loading on metabolic cost, efficiency, and kinematics of roller ski skating

**DOI:** 10.1371/journal.pone.0197592

**Published:** 2018-05-23

**Authors:** Conor M. Bolger, Veronica Bessone, Peter Federolf, Gertjan Ettema, Øyvind Sandbakk

**Affiliations:** 1 Centre for Elite Sports Research, Department of Neuromedicine and Movement Science, Norwegian University of Science and Technology, Trondheim, Norway; 2 Department of Mechanical and Aerospace Engineering, Politecnico di Torino, Torino, Italy; 3 Department of Biomechanics in Sports, Technical University of Munich, Munich, Germany; 4 Department of Sport Science, University of Innsbruck, Innsbruck, Austria; University of Debrecen, HUNGARY

## Abstract

The purpose of the present study was to examine the influence of increased loading of the roller ski on metabolic cost, gross efficiency, and kinematics of roller ski skating in steep and moderate terrain, while employing two incline-specific techniques. Ten nationally ranked male cross-country skiers were subjected to four 7-minute submaximal intervals, with 0, 0.5, 1.0, and 1.5 kg added beneath the roller-ski in a randomized order. This was done on two separate days, with the G2 skating at 12% incline and 7 km/h speed and G3 skating at 5% incline and 14 km/h speed, respectively. At 12% incline, there was a significant increase in metabolic rate and a decrease in gross efficiency with added weight (*P*<0.001 and *P* = 0.002). At 5% incline, no change in metabolic rate or gross efficiency was found (*P* = 0.89 and *P* = 0.11). Rating of perceived exertion (RPE) increased gradually with added weight at both inclines (*P*>0.05). No changes in cycle characteristics were observed between the different ski loadings at either incline, although the lateral and vertical displacements of the foot/skis were slightly altered at 12% incline with added weight. In conclusion, the present study demonstrates that increased loading of the ski increases the metabolic cost and reduces gross efficiency during steep uphill roller skiing in G2 skating, whereas no significant effect was revealed when skating on relatively flat terrain in G3. Cycle characteristics remained unchanged across conditions at both inclines, whereas small adjustments in the displacement of the foot coincided with the efficiency changes in uphill terrain. The increased RPE values with added ski-weight at both inclines indicates that other factors than those measured here could have influenced effort and/or fatigue when lifting a heavier ski.

## Introduction

Cross-country skiing is performed on varying terrain while employing different sub-techniques of the classical or skating styles. In both cases, approximately 50% of the total time is spent racing uphill where skiers perform more work for a given metabolic cost [1–3]. Thus, the most pronounced differences in performance are also observed on uphill sections where energy delivery capacity together with gross efficiency, the ratio of work rate to energy expenditure, are regarded the main determinants of the performance levels of skiers [1, 4, 5].

The importance of skiing efficiency for an athletes’ performance probably reflects the movement’s technical complexity; there are numerous degrees of freedom with respect to the timing of force generation by both the arms and legs [6]. Most often, cycle length is used to distinguish skiers of different performance levels in skating, and any change in technique and/or equipment may not only influence cycle length and rate, but also gross efficiency, and performance [7, 8].

If all other factors are kept constant, the weight of cross-country skiing equipment would discernibly influence the metabolic cost of skiing and performance, in particular on uphill terrain where any additional weight will require extra work against gravity to be done [9]. Moreover, additional weight placed at the periphery (e.g. ski, shoe, and poles) may induce exponentially more cost than a comparable weight placed near the center of mass due to the effects on the large range of lower-limb movement relative to the body’s center of mass [10, 11]. In running, an increase in submaximal oxygen uptake with increased shoe weight was linked to an increased cycle frequency and shorter cycle length [12–14]. However, though oxygen uptake increased as an effect of loading, the gross efficiency may remain constant if the changes in work rate due to added weight coincides with the changes in metabolic rate.

In cross-country skiing, equipment continually becomes lighter, due to new materials and innovative designs, over the years. However, there are still weight differences of more than 5% between the different types of skis and shoes used by top athletes. In addition, skiers train half of the year on different types of roller skis that may vary substantially in weight and are normally heavier than the cross-country skis used in competitions on snow. Although this highlights the existence of variation of equipment weight in cross-country skiing, little is currently known about the quantitative relationship between the weight of the ski/binding and its effect on technique, skiing efficiency, and performance in cross-country skiing. The establishment of such relationship is imperative because both an increase or reduction of weight has an economic cost imposed on the skier. Such evidence is also interesting for understanding how the extra weight of measurement systems placed on the skis, which is often done in research [15, 16], influences skiing technique and efficiency.

Therefore, the purpose of the present study was to examine the influence of increased loading of the roller ski on metabolic cost, gross efficiency, and kinematics of roller ski skating in both steep and moderate terrain while employing two terrain-dependent sub-techniques. We hypothesize that increased distal loading will have a negative impact on the metabolic cost and efficiency both on steep and moderate uphill terrain when skating, although we expect the largest impact in the steep terrain. In addition, we hypothesize that shorter cycle lengths and higher cycle rates will be related to the magnitude of added distal weight.

## Materials and methods

### Participants

Ten male national and international levels cross-country skiers (male, age: 21–28; height: 1.88 ± 0.03 m; body mass: 81.2 ± 7.0 kg; International Ski Federation (FIS) points: 90 ± 37 [mean ± SD]) volunteered to participate in the study. All participants signed an informed consent form before the experiment and were made aware that they could withdraw from the study at any point without providing an explanation. The study was approved by the Norwegian Data Protection Authority and was conducted in accordance with the Declaration of Helsinki.

### Skiing equipment and technique

All athletes used their personal ski boots and poles (with a length ~90% of body height). Total equipment mass was 3.7 ± 0.1 kg, including the kg 2.05 kg roller skis and the remaining weight due to shoes and poles. To minimize variations in rolling resistance, all skiers used the same pair of roller skis (IDT Sports, Lena, Norway). Lead bars weighing 0.25 kg were added to the underside of the roller ski in increments to add appropriate weight according to the protocol described in detail below.

The main idea in our approach was to test skiers at two distinct inclines, while allowing the skiers to choose the most efficient technique while roller skiing. However, we chose inclines where all skiers were expected to use the same sub-technique. Specifically, the G2 and G3 skating techniques were used during this research project; The G2 skating technique (also referred to as offset and V1 skate) is used in steep uphill terrain and involves an asymmetrical double poling action together with every second leg push. Hence, a strong side with synchronized double poling and a leg push-off, as well as a weak side where only the legs push-off is executed. The G3 skating technique (also referred to as V2, 2-skate, and double dance) is used in slight to moderate uphill terrain and involves a symmetrical double poling action together with a single leg push on each side.

### Test protocol

After body-weight measurement and marker placement, the athletes performed a 20-minute low-intensity warm-up and treadmill familiarization, followed by four 7-minute submaximal intervals. The intervals were performed either with normal-unloaded ski equipment (0 kg) or with the addition of 0, 0.5, 1.0, and 1.5 kg evenly distributed below the whole roller skis in blinded randomized order. The addition or removal of weight was done by the test leader and not observed by the athlete, and the skis were placed on the athlete prior each interval by the test leader. On two separate days, the steep (12%) and moderate (5%) terrain techniques were tested at 7 km/h and 14 km/h, respectively. The speed and incline were matched to obtain comparable work rates and to assure that all skiers used G2 at 12% incline and G3 at 5% incline as their self-chosen techniques. The duration and exercise intensity were set to ensure submaximal aerobic steady state occurred.

### Instrumentation and measurement procedure

Ventilatory parameters were assessed by employing open-circuit indirect calorimetry with an Oxycon Pro apparatus (Jaeger GmbH, Hoechberg, Germany) for two minutes at the end of each interval. Prior to each measurement, the VO_2_ and VCO_2_ analyzers were calibrated using a known mixture of gases (16.00 ± 0.04% O_2_ and 5.00 ± 0.1% CO_2_, Riessner-Gase GmbH & Co., Lichtenfels, Germany) and the expiratory flow meter calibrated with a 3-L syringe (Hans Rudolph Inc., Kansas City, MO). Heart rate (HR) was recorded throughout the entirety of each test using the skier's own heart rate monitors. Blood lactate concentration was analyzed using the Biosen C-Line Sport lactate measurement system (EKF Industrial Electronics, Magdeburg, Germany) from 5-μL of fingertip blood collected at the end of each interval. Rating of perceived exertion (RPE) was assessed immediately after each stage. During the four interval trials, three-dimensional movement data were captured from a ten-camera Qualisys Oqus system (Qualisys AB, Gothenburg, Sweden) with a sampling rate of 250 Hz. Retro-reflective markers were placed on the lateral epicondyle, malleolus, and on the boots in positions correspondent to malleolus and toe, on both body sides.

### Gross efficiency

Work rate was calculated as the sum of power against gravity (P_g_ = m · g · sin α · v) and friction (P_f_ = m · g · cos α · μ · v), where m is the body mass of the skier (including equipment and additional weight for each interval), g is the gravitational acceleration, α is the angle of treadmill incline, v is the speed of the treadmill belt, and μ is the frictional coefficient [5, 17]. The rolling friction force (F_f_) of the skis was determined prior to each test day by using a towing test, while the friction coefficient (0.026) was calculated by dividing the friction force by the normal force ((F_n_): μ = F_f_ · F_n_^-1^). The metabolic rate was calculated as the product of VO_2_ and the oxygen energetic equivalent using the associated respiratory exchange ratio and standard conversion tables [18]. Gross efficiency was calculated as the work rate divided by the metabolic rate and presented as a percentage.

### Cycle kinematics

The ski cycle was defined from ski lift-off to the successive ski lift-off. This was determined from the vertical displacement of retro-reflective marker placed on the lateral epicondyle of the skier (placed on the boot). These points served as trigger points, marking the beginning and end of each skate cycle for both G2 and G3 techniques. For each interval, 10 skating cycles were extracted for the analysis of cycle kinematics.

The ski-cycle consists of the lift-off, recovery, and ground contact phases (i.e. gliding and push-off). The lift-off time is the time necessary for the foot to reach the highest point (vertical displacement) within the cycle, while the recovery time is the time it takes for the foot to travel from the highest point until the ski is planted onto the ground. The ground contact time was defined as the time between ski plant and ski lift-off.

The analyzes were performed using custom-written MatlabTM (The MathWorks Inc., Version R2014a, Natwick, MA, USA) codes. The skis’ position in time was re-sampled to 500 data points in order to compare the four weighted conditions and ten participants. The cycle rate was calculated as the number of cycles per second.

### Statistical analysis

**Fig 1:**
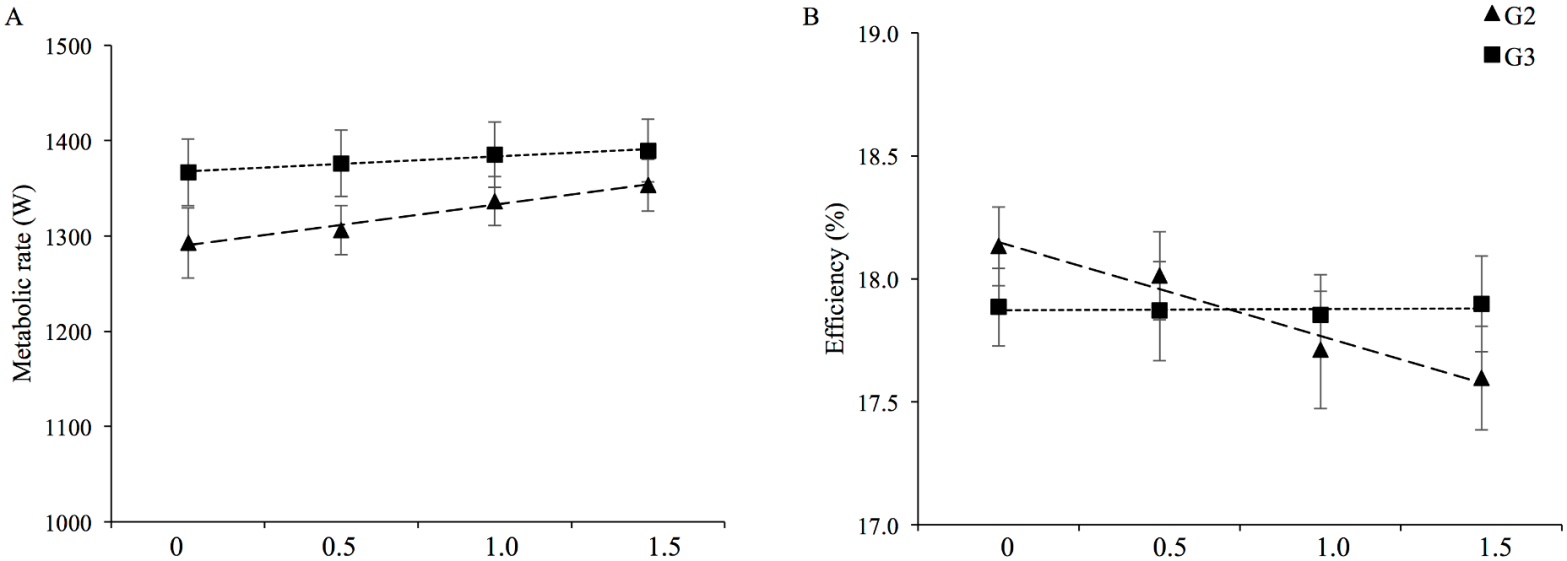
Effect of added weight on efficiency. Metabolic rate (A) and gross efficiency (B) for normal (0 kg, no added weight) and loaded (0.5, 1.0, and 1.5 kg) conditions for 10 elite cross-country skiers while roller skiing at steep (G2 at 12% incline) and moderate (G3 at 5% incline) slopes in the skating technique (mean ± SEM).

All data were presented as mean ± SD in tables and as mean ± SEM in Fig 1. To examine the effects of distal loading within inclines and differences of loading between inclines, a marginal model (population-averaged model) was performed using the Statistical Package for Social Sciences 11.0 (SPSS Inc., Chicago, Illinois, USA). A repeated statement is used to specify covariance structures for longitudinal data on participants, where compound symmetry was used in fitting covariance structure for the residuals across techniques. For fixed effects (regression coefficients) we entered technique and weight (with an interaction term) into the model in order to describe the relationship between the dependent variable and predictor variables for the entire population. P-values < 0.05 were considered statistically significant.

## Results

### Physiological responses and cycle characteristics

The metabolic rate and gross efficiency for unloaded and three loaded conditions at 12% and 5% inclines are documented in Fig 1. At 12% incline, the mean increase in metabolic rate per Watt increase in work rate (i.e. per 0.5 kg increase in weight) was 42.9 ± 31.3 W (*P* < 0.001). Similarly, the mean decrease in efficiency per Watt increase in work rate was 0.2 ± 0.2% (*P* = 0.002). No significant change in metabolic rate (*P* = 0.89) or gross efficiency (*P* = 0.11) with increasing work rate at 5% incline occurred. The interaction between incline and added weight on metabolic rate and gross efficiency was statistically significant (*P* = 0.042 and *P* = 0.022, respectively). The original data are found in the supporting data S1 File.

For 12% and 5% incline, the mean increase in RPE (rated perceived exertion) per Watt increase in work rate was 1.0 ± 1.1 and 0.7 ± 1.1 points, respectively (*P* = 0.007 and *P* = 0.038). There was a statistical trend of increased loading condition on increasing blood lactate concentration at 12% incline (*P* = 0.07) (Table 1). However, blood lactate concentration at 5% incline and heart rate at both inclines did not change significantly.

**Table 1:** Work rate, blood lactate concentration (BLa), heart rate, respiratory exchange ratio (RER) and rated perceived exertion (RPE) for 10 elite cross-country skiers while roller skiing at steep (G2 at 12% incline) and moderate (G3 at 5% incline) inclines while skating with normal (0) or loaded (0.5, 1.0, and 1.5 kg) conditions (mean ± SD).

Variable	Incline	0 kg	0.5 kg	1.0 kg	1.5 kg
Work Rate [W]	12%	234 ± 19	235 ± 19	237 ± 19	238 ± 19
	5%	245 ± 21	246 ± 21	248 ± 21	249 ± 21
BLa [mmol/L]	12%	2.0 ± 0.6	2.1 ± 0.8	2.2 ± 0.9	2.5 ± 1.2
	5%	2.8 ± 1.1	3.3 ± 1.0	3.3 ± 1.4	3.4 ± 1.8
Heart rate [bpm]	12%	157 ± 8	158 ± 8	159 ± 9	160 ± 10
	5%	163 ± 6	164 ± 8	165 ± 10	164 ± 9
RER	12%	0.88 ± 0.04	0.89 ± 0.05	0.89 ± 0.05	0.91 ± 0.07
	5%	0.94 ± 0.10	0.94 ± 0.10	0.94 ± 0.08	0.96 ± 0.11
RPE	12%	13.1 ± 1.5	13.9 ± 1.3*	14.1 ± 1.4*	14.5 ± 1.4*
	5%	12.8 ± 1.5	13.1 ± 1.8*	13.9 ± 1.9*	14.5 ± 1.9*

*Significantly different from 0 kg (*P* < 0.05).

### Kinematics

There was no significant effect of distal loading on cycle rate (or length), lift off time, recovery time, or ground contact time in either the 12% or 5% incline (Table 2).

**Table 2:** Cycle rate (CR), lift-off time (LOT), recovery time (RT), and ground contact time (GCT) for 10 elite cross-country skiers while roller skiing at steep (G2; 12%) and moderate (G3; 5%) inclines while skating with normal (0) or loaded (0.5, 1.0, and 1.5 kg) conditions (mean ± SD). The asymmetrical G2 technique is displayed as strong (S) and weak (W) sides.

Variable	Incline		0 kg	0.5 kg	1.0 kg	1.5 kg
CR [Hz]	12%		0.69 ± 0.03	0.69 ± 0.03	0.68 ± 0.03	0.69 ± 0.03
	5%		0.48 ± 0.03	0.48 ± 0.03	0.48 ± 0.03	0.48 ± 0.03
LOT [s]	12%	S	0.21 ± 0.03	0.21 ± 0.03	0.21 ± 0.03	0.21 ± 0.03
		W	0.24 ± 0.08	0.24 ± 0.08	0.24 ± 0.08	0.25 ± 0.08
	5%		0.24 ± 0.03	0.24 ± 0.03	0.23 ± 0.03	0.24 ± 0.02
RT [s]	12%	S	0.20 ± 0.02	0.18 ± 0.03	0.19 ± 0.01	0.19 ± 0.02
		W	0.27 ± 0.03	0.29 ± 0.03	0.27 ± 0.03	0.27 ± 0.03
	5%		0.49 ± 0.07	0.50 ± 0.07	0.50 ± 0.07	0.51 ± 0.08
GCT [s]	12%	S	0.32 ± 0.04	0.31 ± 0.04	0.33 ± 0.04	0.33 ± 0.04
		W	0.29 ± 0.08	0.28 ± 0.08	0.30 ± 0.09	0.29 ± 0.09
	5%		1.18 ± 0.11	1.18 ± 0.10	1.18 ± 0.10	1.18 ± 0.11

**Fig 2:**
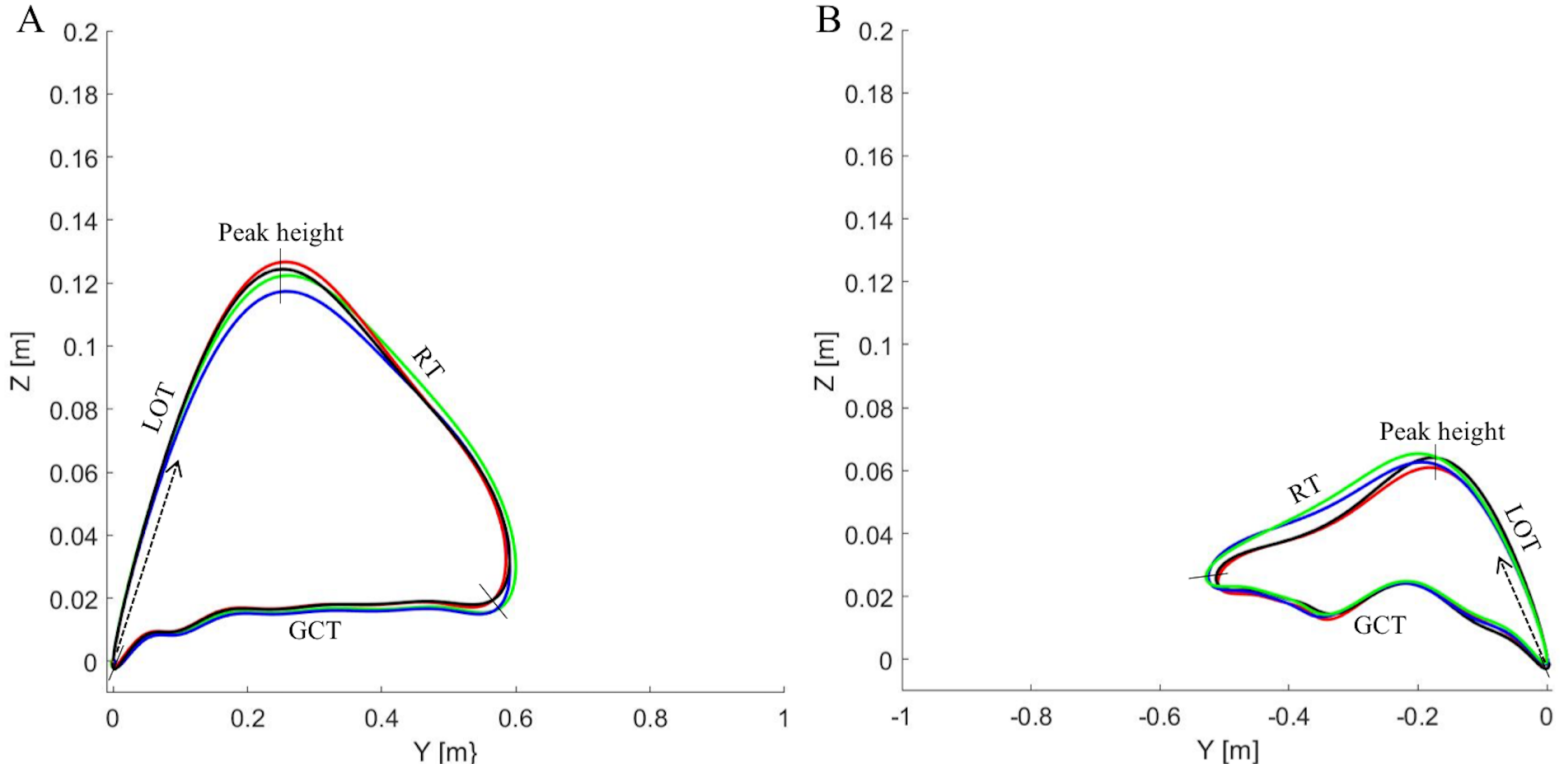
Ski trajectories in G2 skating. The ski trajectories based on the average of 10 consecutive cycles among the 10 skiers in the YZ plane from lift-off to lift-off for the strong (A) and weak (B) sides in the G2 skating technique while roller skiing at 12% incline and 7 km h^-1^. The cycle begins at lift-off (0) and moves in the direction of the arrow, and it contains lift of time (LOT), peak height, recovery time (RT), and ground contact time (GCT). The 0 kg condition is represented by a black line, 0.5 kg by a red, 1.0 kg by a green and 1.5 kg by a blue. The arrow represents the direction of movement starting from lift off (0).

**Fig 3:**
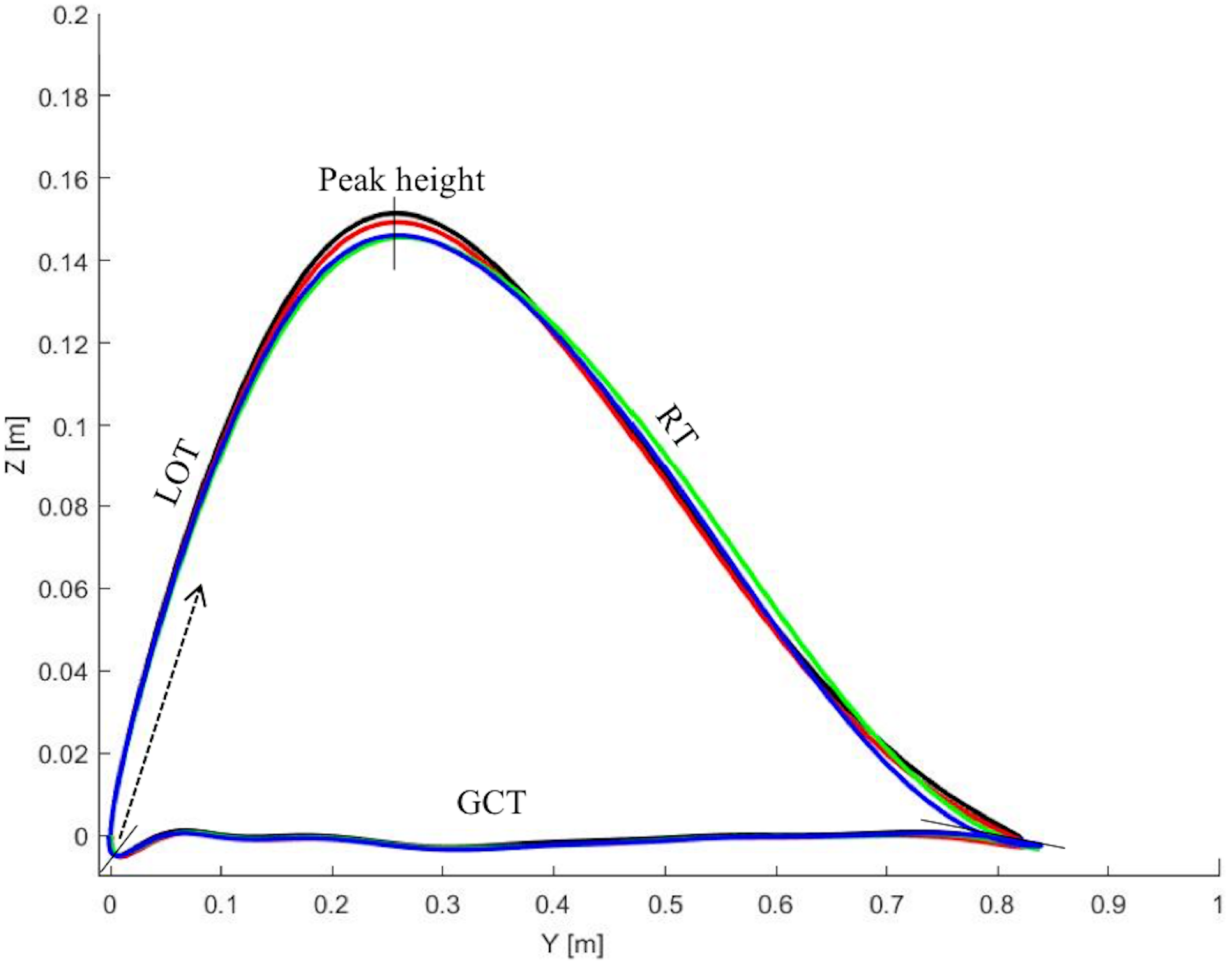
Ski trajectories in G3 skating. The ski trajectories based on the average of 10 consecutive cycles among the 10 skiers in the YZ plane from lift-off to lift-off in the G3 skating technique while roller skiing at 5% incline and 14 km h^-1^. The cycle begins at lift-off (0) and moves in the direction of the arrow and it contains, lift of time (LOT), peak height, recovery time (RT), and ground contact time (GCT). The cycle begins at lift off and contains peak height and ground contact. The 0 kg condition is represented by a black line, 0.5 kg by a red, 1.0 kg by a green and 1.5 kg by a blue.

The trajectory of the foot in the Y (horizontal) and Z (vertical) coordinate plane at 12% incline (strong and weak side) and 5% incline are illustrated in Figs 2 and 3. Individual variations in peak height, ground contact placement are observed, although no significant effects of increased distal loading are present.

Distal loading caused a significant effect on lateral displacement on the weak side (*P* = 0.032), as well as for the vertical displacements of the weak side (*P* = 0.025) and a trend for the strong side (*P* = 0.057) in G2 skating at 12% incline (Table 3). The original data are found in the supporting data S1 File.

**Table 3:** Foot displacement in the vertical, longitudinal, and lateral directions given as a ratio of three distal loads compared to the zero condition for 10 elite cross-country skiers while roller skiing either with the G2 technique at 12% and 7 km h^-1^ or G3 at 5% and 14 km h^-1^, respectively (mean ± SD). The G2 technique is an asymmetrical movement and contains a strong (S) and weak (W) side.

Displacement ratio	Incline		0.5 kg	1.0 kg	1.5 kg
Vertical [Z]	12%	S	0.98 ± 0.10*	1.02 ± 0.09*	1.06 ± 0.06*
		W	0.98 ± 0.08	0.94 ± 0.06	0.97 ± 0.08
	5%		1.02 ± 0.12	1.07 ± 0.18	1.05 ± 0.14
Longitudinal [X]	12%	S	1.03 ± 0.04	0.99 ± 0.04	1.02 ± 0.05
		W	1.02 ± 0.04	0.98 ± 0.07	1.00 ± 0.05
	5%		1.03 ± 0.06	1.03 ± 0.07	1.02 ± 0.05
Lateral [Y]	12%	S	1.04 ± 0.05	1.09 ± 0.08	1.08 ± 0.07
		W	1.05 ± 0.07*	1.02 ± 0.06*	1.01 ± 0.05*
	5%		1.04 ± 0.05	1.05 ± 0.07	1.04 ± 0.06

*Significantly different from 0 kg added weight (P < 0.05).

## Discussion

The purpose of the current study was to examine the influence of increased loading of the roller ski on metabolic cost, gross efficiency, and kinematics of roller ski skating in steep and moderate terrain. When roller skiing on 12% incline with the G2 skating technique, we found an increase in metabolic rate and decreased gross efficiency with added distal weight, whereas in G3 skating at 5% incline no changes occurred. RPE values increased progressively with added weight at both inclines. However, while our findings confirmed the hypothesis that the effect of added distal weight would be most pronounced at the 12% incline, the lack of effect at 5% incline was unexpected. The same applies to the lack of changes in cycle characteristics found in both inclines, since we hypothesized that cycle rate would increase. The only technique changes coinciding with the reduced efficiency at 12% incline with added weight was due to the larger lateral and vertical displacement of the weak side ski.

Although greater metabolic costs and reduced gross efficiency was found with 12% uphill skating, the distal addition of weight did not involve any changes in cycle length, cycle rate or other temporal patterns (i.e., push-off, gliding or swing times) in any of the conditions, though as much as 0.75 kg were added to each ski. This is not in accordance with previous studies performed in running, where it was shown that increased distal loading of the foot resulted in an increase in the cost of the movement [19], which was likely due to increased cycle rate [12–14]. In fact, a recent systematic review and meta-analysis found beneficial effects of lighter shoes on work economy [19]. For example, one study [20] reported that 1 kg extra mass added to the feet reduced cycle rate when running, which was not the case in the present work. However, ski skating includes a different movement pattern than running; in skating the skiers are gliding forward during the entire cycle, even during the push-off phase, whereas running has much larger accelerations and decelerations of the foot during the cycle. In running, the foot has a complete stop in the push-off phase, which is not the case in ski skating. As a result, the acceleration of the foot with respect to the body’s center of mass will also be different for skating and for running.

Also loading close to the center of mass in biathlon, caused by the addition of the rifle (i.e. 3.5 kg added on the back, close to center of mass), led to higher cycle rate and shorter cycle length coinciding with increased metabolic cost in both G2 and G3 skating at comparable inclines to our study [21]. Our observation of unchanged kinematics fits to the preferred movement path paradigm which suggests that the kinematic patterns of highly automatized movements remain unchanged when adapting to equipment modifications [22, 23]. Hence, these highly trained elite skiers may keep their technique unchanged despite the perturbations.

In our case, the lack of modifications in cycle characteristics could theoretically have been caused by the inter-dependence between upper and lower body propulsion when skiing [24–26], meaning that the distribution of propulsive forces from either poles or skis can be altered at the same speed with the same metabolic cost. With the added weight in our case, skiers could have chosen to exert more of the propulsion through poling, which is regarded a highly efficient movement pattern [4, 5, 27] and thereby maintained their cycle characteristics without changes in metabolic cost. A possible shift from leg propulsion to more poling should have allowed the skis to travel less in the anterior-posterior direction during the recovery phase, which was not the case in the present investigation.

The fact that skiers rated higher RPE with increased loading not only at 12% incline, but also at 5%, indicates that other factors than those measured here are involved in the skiers’ rating of effort. These greater RPE-values rated at submaximal workloads are as expected and indicates that skiers felt an extra cost associated to the added weight. However, the underlying mechanisms are currently unclear, and could be due to, for example, changes in the loading and activation patterns of local muscles when lifting a heavier ski that was also present at the moderate incline without influencing the overall metabolic cost at this condition or intensity. Through interviews, our skiers also suggested a greater muscular load with added weight and argued that this would also have caused a performance-deficit.

Coinciding with the reduced efficiency in G2 skating at steep uphill, lateral and vertical displacement on the weak side ski decreased. To what extent this was linked to the reduced efficiency was not examined here, though with 0.75 kilograms added to each foot skiers clearly needed to alter the way they skied somehow. However, since this study was performed as steady state roller skiing in a controlled indoor environment, the effects might have been different on other scenarios or conditions. For example, race scenarios include a greater rate of change in kinetic energy through accelerations in the start, attacks during mass-starts and/or with changes in terrain or sub-technique transitions. Furthermore, the importance of skiing at higher speeds and intensities in a performance setting would have an influence and since we did not measure directly performance, care should be taken when interpreting our findings to these settings. However, though the present study showed that distal weight only had moderate effects on G2 skating at 12% incline, we hypothesize that the effects would be larger on performance with a more ecological design where the abovementioned factors are included when skiing outside or while snow skating. Hence, this should be a point of departure for exploring these effects more in detail in follow-up studies.

## Conclusions

The present study demonstrates that added loading of ski increases the metabolic cost and reduces gross efficiency during steep uphill roller skiing, whereas no significant effect was revealed when skating on relatively flat terrain. However, all skiers rated higher effort with added weight in both techniques, which indicates that mechanisms not related to increased metabolic costs at moderate inclines are altered when using heavier skis. For example, changes in the loading and activation patterns of local muscles when lifting a heavier ski without influencing the overall metabolic cost is a likely explanation. However, although the effects of adding weight on the metabolic cost, gross efficiency and kinematic patterns are relatively small, other factors than those measured should be further outlined in future experiments. Although no significant modifications in the cycle characteristics were revealed between conditions at either incline, the addition of distal weight resulted in small adjustments of the technique by more lateral and vertical displacement on the weak side when G2 skating at a steep incline to accommodate the increased distal loading and sustain cycle rate.

## Supporting information

S1 File Excel file containing data presented in the results chapter. (XLSX)

## References

[1] Sandbakk Ø, Ettema G, Leirdal S, Jakobsen V, Holmberg H-C. Analysis of a sprint ski race and associated laboratory determinants of world-class performance. European Journal of Applied Physiology. 2011;111(6):947–57. doi: 10.1007/s00421-010-1719-9 21079989 10.1007/s00421-010-1719-9 PMC3092926

[2] Bolger CM, Kocbach J, Hegge AM, Sandbakk Ø. Speed and Heart-Rate Profiles in Skating and Classical Cross-Country-Skiing Competitions. International Journal of Sports Physiology and Performance. 2015;10(7):873–80. doi: 10.1123/ijspp.2014-0335 25671845 10.1123/ijspp.2014-0335

[3] Sandbakk Ø, Losnegard T, Skattebo Ø, Hegge AM, Tønnessen E, Kocbach J. Analysis of classical time-trial performance and technique-specific physiological determinants in elite female cross-country skiers. Frontiers in Physiology. 2016;7. 10.3389/fphys.2016.00326 PMC4971020 27536245

[4] Sandbakk Ø, Holmberg H-C, Leirdal S, Ettema G. Metabolic rate and gross efficiency at high work rates in world class and national level sprint skiers. European Journal of Applied Physiology. 2010;109(3):473–81. doi: 10.1007/s00421-010-1372-3 20151149 10.1007/s00421-010-1372-3

[5] Sandbakk Ø, Hegge AM, Ettema G. The role of incline, performance level, and gender on the gross mechanical efficiency of roller ski skating. Frontiers in Physiology. 2013;4. 10.3389/fphys.2013.00293 PMC3804929 24155722

[6] Sandbakk Ø, Holmberg H-C. A reappraisal of success factors for Olympic cross-country skiing. International Journal of Sports Physiology and Performance. 2014;9(1):117–21. doi: 10.1123/ijspp.2013-0373 24088346 10.1123/ijspp.2013-0373

[7] Bilodeau B, Rundell KW, Roy B, Boulay MR. Kinematics of cross-country ski racing. Medicine and Science in Sports and Exercise. 1996;28(1):128–38. 8775365 10.1097/00005768-199601000-00024

[8] Sandbakk Ø, Holmberg H-C. Physiological Capacity and Training Routines of Elite Cross-Country Skiers: Approaching the Upper Limits of Human Endurance. International Journal of Sports Physiology and Performance. 2017:1–26. 10.1123/ijspp.2016-0749 28095083

[9] Moxnes JF, Sandbakk Ø, Hausken K. Using the power balance model to simulate cross-country skiing on varying terrain. Open Access Journal of Sports Medicine. 2014;5:89 doi: 10.2147/OAJSM.S53503 24891815 10.2147/OAJSM.S53503 PMC4019618

[10] Myers M, Steudel K. Effect of limb mass and its distribution on the energetic cost of running. Journal of Experimental Biology. 1985;116(1):363–73. 4056656 10.1242/jeb.116.1.363

[11] Schertzer E, Riemer R. Metabolic rate of carrying added mass: a function of walking speed, carried mass and mass location. Applied Ergonomics. 2014;45(6):1422–32. doi: 10.1016/j.apergo.2014.04.009 24793822 10.1016/j.apergo.2014.04.009

[12] Lussiana T, Fabre N, Hébert-Losier K, Mourot L. Effect of slope and footwear on running economy and kinematics. Scandinavian Journal of Medicine & Science in Sports. 2013;23(4). 10.1111/sms.12057 23438190

[13] Cureton KJ, Sparling PB. Distance running performance and metabolic responses to running in men and women with excess weight experimentally equated. Medicine & Science in Sports & Exercise. 1980;12(4):288–94. 7421479

[14] Frederick E. Physiological and ergonomics factors in running shoe design. Applied Ergonomics. 1984;15(4):281–7. 15676526 10.1016/0003-6870(84)90199-6

[15] Göpfert C, Pohjola MV, Linnamo V, Ohtonen O, Rapp W, Lindinger SJ. Forward acceleration of the centre of mass during ski skating calculated from force and motion capture data. Sports Engineering. 2017;20(2):141–53.

[16] Hoset M, Rognstad A, Rølvåg T, Ettema G, Sandbakk Ø. Construction of an instrumented roller ski and validation of three-dimensional forces in the skating technique. Sports Engineering. 2014;17(1):23–32.

[17] De Koning JJ, Foster C, Lampen J, Hettinga F, Bobbert MF. Experimental evaluation of the power balance model of speed skating. Journal of Applied Physiology. 2005;98(1):227–33. doi: 10.1152/japplphysiol.01095.2003 15591304 10.1152/japplphysiol.01095.2003

[18] Peronnet F, Massicotte D. Table of nonprotein respiratory quotient: an update. Canadian Journal of Sport Science. 1991;16(1):23–9. 1645211

[19] Fuller JT, Bellenger CR, Thewlis D, Tsiros MD, Buckley JD. The Effect of Footwear on Running Performance and Running Economy in Distance Runners. Sports Med. 2015; 45(3): 411–422. doi: 10.1007/s40279-014-0283-6 25404508 10.1007/s40279-014-0283-6

[20] Martin PE. Mechanical and physiological responses to lower extremity loading during running. Medicine and Science in Sports and Exercise. 1985;17(4):427–33. 4033398 10.1249/00005768-198508000-00004

[21] Stöggl T, Bishop P, Höök M, Willis S, Holmberg H-C. Effect of carrying a rifle on physiology and biomechanical responses in biathletes. Medicine and Science in Sports and Exercise. 2015;47(3):617–24. doi: 10.1249/MSS.0000000000000438 25003775 10.1249/MSS.0000000000000438

[22] Nigg B. Muscle tuning and preferred movement path-a paradigm shift. Current Issues in Sport Science (CISS). 2017; 2:007.

[23] Nigg BM, Vienneau J, Smith AC, Trudeau MB, Mohr M, Nigg SR. The Preferred Movement Path Paradigm: Influence of Running Shoes on Joint Movement. Medicine & Science in Sports & Exercise. 2017; 49(8):1641–1648. 28277405 10.1249/MSS.0000000000001260

[24] Grasaas CÅ, Ettema G, Hegge AM, Skovereng K, Sandbakk Ø. Changes in technique and efficiency after high-intensity exercise in cross-country skiers. International Journal of Sports Physiology and Performance. 2014;9(1):19–24. doi: 10.1123/ijspp.2013-0344 23982869 10.1123/ijspp.2013-0344

[25] Leirdal S, Sandbakk Ø, Ettema G. Effects of frequency on gross efficiency and performance in roller ski skating. Scandinavian Journal of Medicine & Science in Sports. 2013;23(3):295–302. 22092985 10.1111/j.1600-0838.2011.01379.x

[26] Sandbakk Ø, Ettema G, Holmberg H-C. The physiological and biomechanical contributions of poling to roller ski skating. European Journal of Applied Pphysiology. 2013;113(8):1979–87. 10.1007/s00421-013-2629-4 23543069

[27] Sandbakk Ø, Ettema G, Holmberg H-C. The influence of incline and speed on work rate, gross efficiency and kinematics of roller ski skating. European Journal of Applied Physiology. 2012;112(8):2829–38. doi: 10.1007/s00421-011-2261-0 22127680 10.1007/s00421-011-2261-0

